# Otoprotective Effects of Zingerone on Cisplatin-Induced Ototoxicity

**DOI:** 10.3390/ijms21103503

**Published:** 2020-05-15

**Authors:** Chang Ho Lee, Da-hye Lee, So Min Lee, So Young Kim

**Affiliations:** Department of Otorhinolaryngology, CHA University College of Medicine, Seongnam 13496, Korea; hearwell@gmail.com (C.H.L.); ldada72@naver.com (D.-h.L.); lws6812@naver.com (S.M.L.)

**Keywords:** hearing loss, cisplatin, zingerone, cytochrome P450, tumor necrosis factor alpha

## Abstract

Previous studies have described the effects of zingerone (ZO) on cisplatin (CXP)-induced injury to the kidneys, liver, and other organs but not to the cochlea. This study aimed to investigate the effects of ZO on CXP-induced ototoxicity. Eight-week-old Sprague–Dawley rats were used and divided into a control group, a CXP group, and a CXP + ZO group. Rats in the CXP group received 5 mg/kg/day CXP intraperitoneally for five days. Rats in the CXP + ZO group received 5 mg/kg/day CXP intraperitoneally for five days and 50 mg/kg/day ZO intraperitoneally for seven days. Auditory brainstem response thresholds (ABRTs) were measured before (day 0) and after (day 10) drug administration. Cochlear histology was examined using hematoxylin and eosin (H&E) staining and cochlear whole mounts. The expression levels of *cytochrome P450 (CYP)1A1, CYP1B1, inducible nitric oxide synthase (iNOS), nuclear factor kappa B (NFκB), tumor necrosis factor alpha (TNFα)*, and *interleukin 6 (IL6)* were estimated using quantitative reverse transcription-polymerase chain reaction. The expression levels of *heme oxygenase 1 (HO1)* and caspase 3 were analyzed via Western blotting. The auditory thresholds at 4, 8, and 16 kHz were attenuated in the CXP + ZO group compared with the CXP group. The mRNA expression levels of *CYP1A1*, *CYP1B1*, *iNOS*, *NFκB*, *TNFα*, and *IL6* were lower in the CXP + ZO group than in the CXP group. The protein expression levels of *HO1* and *caspase 3* were lower in the CXP + ZO group than in the CXP group. Cotreatment with ZO exerted otoprotective effects against CXP-induced cochlear injury via antioxidative and anti-inflammatory activities involving *CYPs*, *iNOS*, *NFκB*, and *TNFα*.

## 1. Introduction

Oxidative stress combined with inflammation in the cochlea is one of the main pathophysiologies of cochlear hearing loss [1,2,3]. Noise-induced hearing loss, age-related hearing loss, and hearing loss induced by ototoxic drugs, including aminoglycosides and anticancer agents, have been reported to be mediated by oxidative stress and inflammation in cochlear hair cells and spiral ganglion cells [1,2,3]. Cisplatin (CXP)-induced ototoxicity is accompanied by increases in the levels of reactive oxygen species; the inflammatory molecules *tumor necrosis factor alpha (TNFα), interleukin 1β (IL1β)*, and *interleukin 6 (IL6)*; and *nuclear factor kappa B (NFκB)* [4,5,6]. CXP treatment increases the expression of *NFκB* and *inducible nitric oxide synthase (iNOS)* in the cochlea, especially in the stria vascularis and spiral ligament [4]. Activation of *NFκB* increases the levels of proinflammatory cytokines, such as *TNFα*, *IL1β*, and *IL6*, in the cochlea [7]. These oxidative stress responses and inflammatory responses induce apoptosis activation by activating caspase 3 [8]. The sensory hair cells of the organ of Corti, the stria vascularis, and spiral ganglion cells have been reported to be the main targets of CXP-induced oxidative and inflammatory damage [9,10]. Animal studies have shown that these ototoxic injuries are initiated by the degeneration and loss of cochlear outer hair cells and are accompanied by neuronal death and spiral ganglion cell degeneration. This histological damage is associated with shifts in the auditory brainstem response (ABR) threshold (ABRT) and optoacoustic emissions, which have been characterized as dose-dependent, and with irreversible hearing loss [10,11]. A number of in vivo and in vitro studies have suggested treatment with preservative agents to limit CXP-induced ototoxic injury [8,12,13,14]. However, the clinical use of these agents has been impeded by the questionable and potentially toxic effects of human doses.

Zingerone (ZO; 4-para methoxy-4-hydroxyphenyl-2-butanone) has antioxidant, anti-inflammatory, and antiapoptotic effects [15] that are presumed to be related to its protective effects against radiation-induced intestinal injury [16], lipopolysaccharide-induced liver damage [17], and sepsis-triggered nephrotoxicity [18]. Moreover, ZO has protective effects against CXP-induced toxicities, such as nephrotoxicity [15,19], hepatotoxicity [20], cardiotoxicity [21], and ovarian and uterine toxicity [22]. Because the molecular mechanisms underlying CXP-induced nephrotoxicity overlap with those underlying CXP-induced ototoxicity [23], it would be reasonable to expect that ZO has otoprotective effects against CXP-induced ototoxicity. However, to the best of our knowledge, no study has investigated the protective effect of ZO against ototoxicity.

Here, we hypothesized that the antioxidative, anti-inflammatory, and antiapoptotic effects of ZO might be effective against CXP-induced ototoxicity. To test this hypothesis, adult rats were cotreated with ZO during exposure to CXP. We used the dose of ZO reported in previous studies on nephrotoxicity [19]. We simultaneously pretreated rats with both ZO and CXP instead of with only ZO, as was done in previous studies [19]. The results demonstrated preservation of auditory function and cochlear histology in cochlear outer hair cells and spiral ganglion cells. In addition, the increases in the expression levels of *iNOS*, *NFκB*, the proinflammatory molecules *TNFα* and *IL6*, and caspase 3 were reduced in the ZO-treated group. The present results broaden the application of ZO to ototoxicity and suggest a new approach to limit CXP-induced ototoxic injury.

## 2. Results

**Figure 1:**
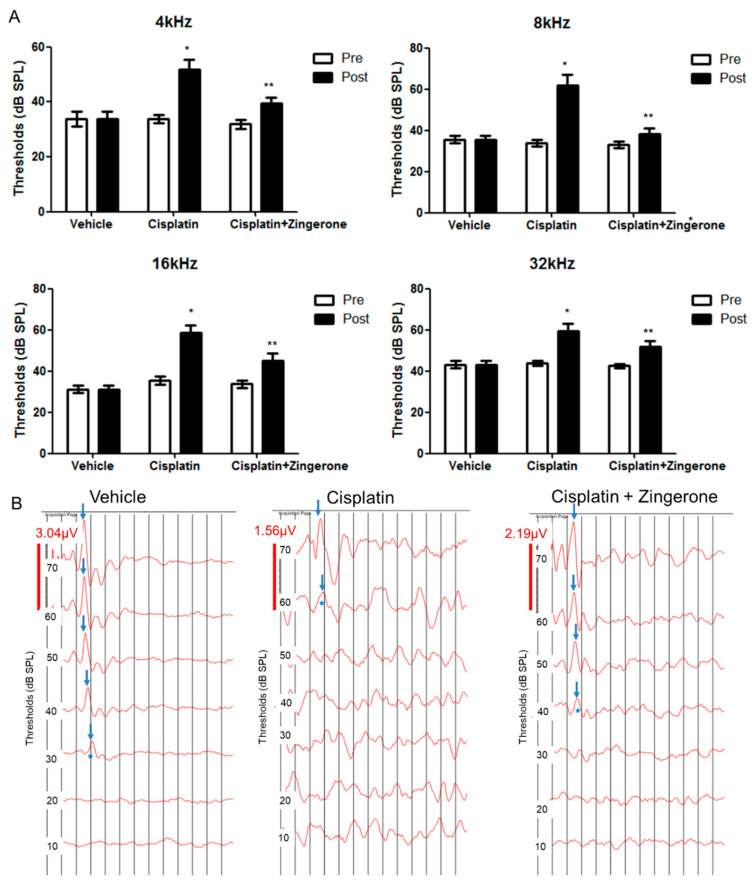
Auditory brainstem response (ABR) thresholds on day 0 (pretreatment) and day 10 (post-treatment). (**A**) The ABR thresholds differed among the three groups (* *p* < 0.05 for the control vs. cisplatin groups by repeated measures ANOVA with Tukey’s posthoc test). The ABR thresholds in the cisplatin + zingerone group on day 10 were attenuated compared with those in the cisplatin group (** *p* < 0.05 for the cisplatin vs. cisplatin + zingerone groups by repeated measures ANOVA with Tukey’s posthoc test). The values shown in the graphs are the means ± standard deviations. (**B**) The ABR waveforms at 8 kHz are presented for each group (the arrows indicate wave II; * indicates ABR thresholds).

Auditory thresholds were increased after CXP injection (Figure 1). The changes in auditory thresholds differed significantly among the groups (*p* < 0.001 for both pretreatment and post-treatment and for frequencies of 4, 8, 16, and 32 kHz; repeated measures ANOVA). The auditory threshold was higher in the CXP group than in the control group on day 10 (*p* < 0.001, repeated measures ANOVA with Tukey’s test). The mean auditory thresholds in the CXP group on day 10 were 51.88 (SD = 3.44) decibel sound pressure level (dB SPL), 61.88 (SD = 5.26) dB SPL, 58.75 (SD = 3.40) dB SPL, and 58.75 (SD = 3.75) dB SPL for 4, 8, 16, and 32 kHz, respectively. The auditory thresholds in the CXP + ZO group were lower than those in the CXP group on day 10 (*p* = 0.001, repeated measures ANOVA with Tukey’s test). The mean auditory thresholds in the CXP + ZO group on day 10 were 37.5 (SD = 2.5) dB SPL, 37.5 (SD = 2.81) dB SPL, 43.13 (SD = 3.84) dB SPL, and 50.00 (SD = 3.03) dB SPL for 4, 8, 16, and 32 kHz, respectively.

**Figure 2:**
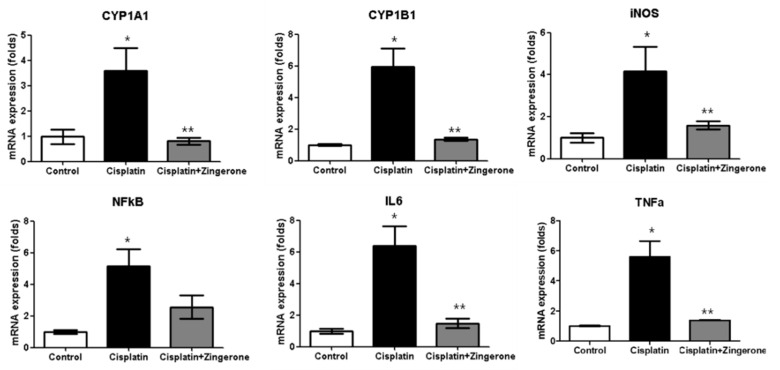
The mRNA expression levels of cytochrome P450 (CYP)1A1, CYP1B1, inducible nitric oxide synthase (iNOS), nuclear factor-κB (NFκB), tumor necrosis factor alpha (TNFα), and interleukin 6 (IL6) were higher in the cisplatin (CXP) group than in the control group (* *p* < 0.05, ANOVA with Tukey’s posthoc test). The cisplatin + zingerone group exhibited lower mRNA expression levels of CYP1A1, CYP1B1, iNOS, NFκB, TNFα, and IL6 than the cisplatin group (** *p* < 0.05, ANOVA with Tukey’s posthoc test). The values shown in the graphs are the means ± standard deviations.

The cochlear mRNA expression levels of *cytochrome P450 (CYP)1A1*, *CYP1B1*, *iNOS*, *NFκB*, *IL6*, and *TNFα* were higher in the CXP group than in the control group, and these increases were reversed in the CXP + ZO group (Figure 2). The *CYP1A1* mRNA levels in the CXP and CXP + ZO groups were 3.59-fold (SD = 0.90) and 0.81-fold (SD = 0.13) higher, respectively, than the level in the control group (*p* = 0.003 with ANOVA, *p* = 0.008 with Tukey’s test for control vs. CXP, and *p* = 0.005 with Tukey’s test for CXP vs. CXP + ZO). The *CYP1B1* mRNA levels in the CXP and CXP + ZO groups were 5.93-fold (SD = 1.18) and 1.35-fold (SD = 0.11) higher, respectively, than the level in the control group (*p* < 0.001 with ANOVA, *p* < 0.001 with Tukey’s test for control vs. CXP, and *p* < 0.001 with Tukey’s test for CXP vs. CXP + ZO). The *iNOS* mRNA levels in the CXP and CXP + ZO groups were 4.16-fold (SD = 1.19) and 1.59-fold (SD = 0.18) higher, respectively, than the level in the control group (*p* = 0.011 with ANOVA, *p* = 0.012 with Tukey’s test for control vs. CXP, and *p* = 0.045 with Tukey’s test for CXP vs. CXP + ZO). The *NFκB* mRNA levels in the CXP and CXP + ZO groups were 5.13-fold (SD = 1.11) and 2.56-fold (SD = 0.73) higher, respectively, than the level in the control group (*p* = 0.004 with ANOVA, *p* = 0.003 with Tukey’s test for control vs. CXP, and *p* = 0.070 for CXP vs. CXP + ZO). The *IL6* mRNA levels in the CXP and CXP + ZO groups were 6.40-fold (SD = 1.21) and 1.47-fold (SD = 0.30) higher, respectively, than the level in the control group (*p* < 0.001 with ANOVA, *p* < 0.001 with Tukey’s test for control vs. CXP and CXP vs. CXP + ZO). The *TNFα* mRNA levels in the CXP and CXP + ZO groups were 5.59-fold (SD = 1.05) and 1.39-fold (SD = 0.02) higher, respectively, than the level in the control group (*p* < 0.001 with ANOVA, *p* < 0.001 with Tukey’s test for control vs. CXP and CXP vs. CXP + ZO).

**Figure 3:**
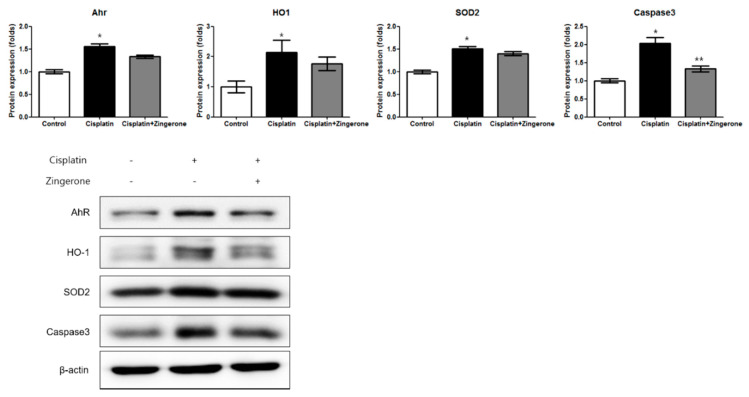
The protein expression levels of the aryl hydrocarbon receptor (AhR), heme oxygenase 1 (HO1), superoxide dismutase 2 (SOD2), and caspase 3 were higher in the cisplatin group than in the control group (* *p* < 0.05, ANOVA with Tukey’s post hoc test, control vs. cisplatin groups; ** *p* < 0.05, ANOVA with Tukey’s post hoc test, cisplatin vs. cisplatin + zingerone groups). The protein expression levels of caspase 3 were lower in the cisplatin + zingerone group than in the cisplatin group (*p* = 0.03, ANOVA with Tukey’s posthoc test). The values shown in the graphs are the means ± standard deviations. (+: not administered, -: administered).

The cochlear protein expression levels of the aryl hydrocarbon receptor (AhR), heme oxygenase 1 (HO1), superoxide dismutase 2 (SOD2), and caspase 3 were higher in the CXP group than in the control group (Figure 3). In the CXP + ZO group, the increase in the expression of AhR and caspase 3 was smaller than that in the CXP group. The AhR protein level was lower in the CXP + ZO group than in the CXP group, but the levels in these groups were 1.44-fold (SD = 0.04) and 1.66-fold (SD = 0.05) higher, respectively, than the level in the control group (*p* < 0.001 with ANOVA, *p* < 0.001 with Tukey’s test for control vs. CXP, and *p* = 0.07 with Tukey’s test for CXP vs. CXP + ZO). The HO1 protein level did not differ significantly between the CXP and CXP + ZO groups, and the levels in these groups were 2.15-fold (SD = 0.40) and 1.77-fold (SD = 0.22) higher, respectively, than that in the control group (*p* = 0.038 with ANOVA, *p* = 0.033 with Tukey’s test for control vs. CXP, and *p* = 0.18 with Tukey’s test for CXP vs. CXP + ZO). The SOD2 protein level did not differ significantly between the CXP and CXP + ZO groups, and the levels in these groups were 1.51-fold (SD = 0.05) and 1.40-fold (SD = 0.11) higher, respectively, than that in the control group (*p* < 0.001 with ANOVA, *p* < 0.001 with Tukey’s test for control vs. CXP, and *p* = 0.206 with Tukey’s test for CXP vs. CXP + ZO). The caspase 3 protein levels were 2.03-fold (SD = 0.16) and 1.33-fold (SD = 0.20) higher in the CXP and CXP + ZO groups, respectively, than in the control group (*p* < 0.001 with ANOVA, *p* < 0.001 with Tukey’s test for control vs. CXP, and *p* = 0.115 with Tukey’s test for CXP vs. CXP + ZO).

**Figure 4:**
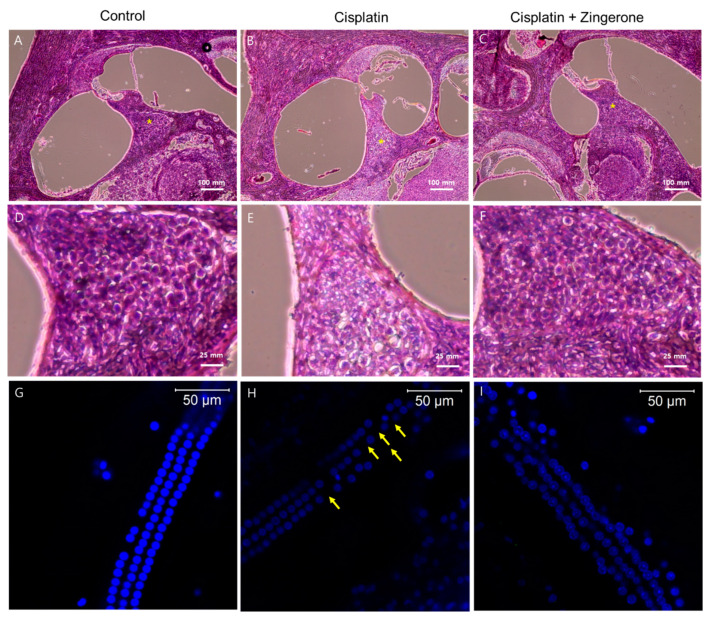
Hematoxylin and eosin (H&E) staining of the cochlea (**A**–**F**) and whole mounts of the cochlear basal turn (**G**–**I**). The control group exhibited intact spiral ganglion cells (**A**) and outer hair cells (**G**). Compared with the cisplatin group (**B**,**E**,**H**), the cisplatin + zingerone group (**C**,**F**,**I**) exhibited a preserved number and arrangement of spiral ganglion cells (* in **A**–**C** and **D**–**F**) and outer hair cells (the arrows indicate the loss of outer hair cells).

Cochlear hematoxylin and eosin (H&E) staining revealed a higher density of spiral ganglion cells in the CXP + ZO group than in the CXP group (Figure 4). Compared with the spiral ganglion cells in the CXP + ZO group, those in the CXP group contained vacuoles with condensed chromatin and were disordered. Cochlear whole mount staining (*n* = 2 per group, for a total of 6 cochleae) demonstrated the loss of outer hair cells in the CXP group, while these cells were preserved in the CXP + ZO group. The loss of outer hair cells was most commonly observed in the basal turn of the cochlea (percentage of outer hair cell survival (mean of both cochleae) = 98.6% (122/124) in the control group, 82.25% (102/124) in the CXP group, and 90.3% (112/124) in the CXP + ZO group).

## 3. Discussion

ZO attenuated CXP-induced ototoxicity in a rat model. The effects of ZO were assessed functionally via measurement of ABRT reduction and morphologically via analysis of cochlear outer hair cell and spiral ganglion cell counts and arrangements. ZO exerted its antioxidative effect by decreasing the expression of *CYP1A1*, *CYP1B1*, and *iNOS*. This alleviation of oxidative stress was linked with attenuated expression of *NFκB* and proinflammatory molecules (*TNFα* and *IL6*). In addition, the decreased apoptotic activity could be predicted by the decreased expression of caspase 3. In summary, the antioxidative, anti-inflammatory, and antiapoptotic effects of ZO on CXP-induced injury occur in the cochlea as well as in the kidneys and other organs. Because ZO is an herbal compound that is used to prevent and treat other diseases and symptoms, it can be used in patients concerned about ototoxic injury resulting from anticancer treatment with CXP. The equivalent dose of ZO in humans is expected to be 3 g in adults (50 mg/kg × 60 kg), which is a feasible dose without definite toxicity. Further study on the clinical application of ZO for ototoxicity is warranted. To our knowledge, this report is the first to describe the effect of ZO on ototoxic injury.

The antioxidative and anti-inflammatory effects of ZO have been described in two CXP-induced nephrotoxicity model studies [15,19]. Both studies used identical doses of ZO (50 mg/kg/day, administered three times) and CXP (7 mg/kg, administered one time) [15,19]. In addition, previous studies have demonstrated the dose-dependent antioxidative and anti-inflammatory effects of ZO and the lack of adverse effects on body weight or kidney function with doses of 10, 20, and 50 mg/kg [19]. Therefore, the dose of ZO used in the present study (50 mg/kg/day, administered three times) was identical to that used in those previous studies. However, the dose of CXP used in this study (5 mg/kg/day, administered three times) was higher than that used in previous studies. Although a higher dose of CXP was used in the current study than in previous studies, the antioxidative and anti-inflammatory effects of ZO were observed in the cochlea.

ZO cotreatment attenuated CXP-induced expression of the CYP enzymes *CYP1A1* and *CYP1B1* in the cochlea. CYP enzymes belong to a superfamily of mono-oxygenases and are involved in oxidative stress responses after CXP-induced injury [24]. A previous CXP-induced hepatotoxicity study in rats also reported attenuation of CYP enzyme expression in the ZO pretreatment group [20]. Because ZO is a polyphenolic alkanone, the phenolic component might play a role as a free radical scavenger and exert cytoprotective effects [25]. In addition, the expression of *iNOS* was reduced in the ZO cotreatment group in the present study. CXP-induced ototoxic injury has been reported to induce the expression of *iNOS*, a major enzyme that generates nitric oxide [26]. Similar to the present study, a few previous studies showed that after ZO treatment, a reduction in *iNOS* expression occurred in other organs, such as the liver [20], ovaries, and uterus [22].

In addition to exerting antioxidative effects, ZO cotreatment reduced the inflammatory response involving *NFκB*, *TNFα*, and *IL6* in the cochlea. *NFκB* participates in mitogen-activated protein kinase signaling cascades, which activate proinflammatory cytokines and chemokines [27]. *NFκB* activation induced by CXP-mediated injury increases the secretion of preformed proinflammatory cytokines, including *TNFα* and *IL6*, without increases in their mRNA or protein expression [7]. This immediate secretion of proinflammatory cytokines was found to occur within 24 h after CXP treatment in an in vitro study [7]. Preformed *TNFα* is reportedly stored in inflammatory cells, such as mast cells and neutrophils [28,29]. Because we cotreated rats with ZO and CXP, these preformed inflammatory cytokines might have been released and might have induced inflammation; this possibility was supported by the elevated expression levels of *TNFα* and *IL6* observed in the CXP + ZO group compared with the control group. *TNFα* has been reported to play a pivotal role in initiating inflammatory responses subsequent to CXP ototoxicity [7]. Blockade of *TNFα* activation by the *TNFα* inhibitor etanercept suppresses the secretion of proinflammatory cytokines, including *IL6*, in the peripheral blood [7]. On the other hand, activation of *TNFα* can activate *NFκB* via phosphorylation of inhibitr of nuclear factor kappa B (IκB) kinase and intranuclear translocation of *NFκB* [30]. By inhibiting the de novo synthesis of *TNFα* and *NFκB*, ZO can terminate the vicious cycles of CXP-induced inflammatory responses.

In the present study, CXP-induced apoptosis related to caspase 3 was attenuated in the cochlea in the ZO cotreatment group, although the caspase 3 level in this group was still higher than that in the control group. The immediate inflammatory and apoptotic responses after CXP administration could not be reversed in this study because ZO and CXP were simultaneously administered; in contrast, in previous studies, ZO was administered before CXP was injected [21,22]. Moreover, although ZO showed protective effects against oxidative stress and inflammation, it could not prevent activation of other apoptotic pathways, such as the intrinsic pathway involving mitochondrial cytochrome c and caspases 9, 6, and 7 [31] or activation of the transient receptor potential vanilloid 1 channel in cochlear hair cells [32]. The combination of another therapy that inhibits these other CXP-induced injuries with ZO might be a promising therapeutic option for CXP-induced ototoxicity.

## 4. Materials and Methods

### 4.1. Animal Treatments

**Figure 5:**
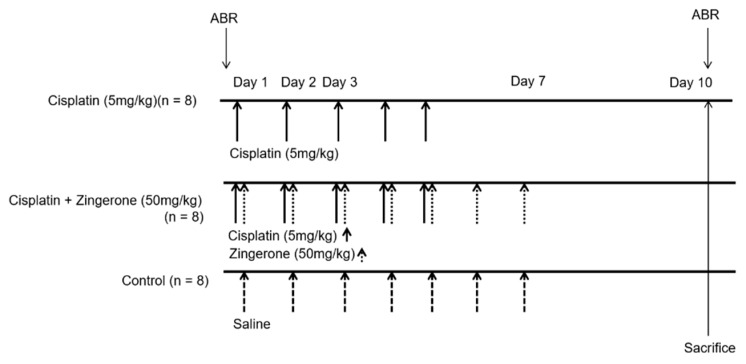
Dosing schedules and measurements used in the present study. Cisplatin (5 mg/kg) was administered to rats in the cisplatin group for 5 days (*n* = 10). Cisplatin (5 mg/kg) was administered for 5 days and zingerone (50 mg/kg) was administered for 7 days to rats in the cisplatin + zingerone group (*n* = 10). An equal volume of saline was administered to rats in the control group (*n* = 10). Auditory brainstem responses (ABRs) were measured on days 0 and 10.

Eight-week postnatal female Sprague–Dawley rats (*n* = 24) were used for experiments. The Institutional Animal Care and Use Committee of CHA University Medical School (IACUC190047, 7 December, 2018) approved the experiments. All experiments were conducted according to the institutional guidelines. Rats were classified into a control group (*n* = 8), CXP group (*n* = 8), or CXP + ZO group (*n* = 8, Figure 5). Rats in the CXP group were administered 5 mg/kg/day CXP intraperitoneally from days 1 to 5. Rats in the CXP + ZO group received 50 mg/kg/day ZO intraperitoneally, immediately after CXP injection, from days 1 to 7. The dose of ZO was determined from previous studies [19,22]. No weight loss or death occurred after ZO administration in this study. Rats in the control group received an identical volume of normal saline by intraperitoneal injection from days 1 to 7.

The ABRTs for a tone burst at 4, 8, 16, or 32 kHz were measured on days 0 and 10, as performed in a previous study [33]. Rats were anesthetized by intraperitoneal injection of a mixture of Zoletil (40 mg/kg) and xylazine (10 mg/kg), and electrodes were inserted at the vertex and the ipsilateral and contralateral retroauricular areas. The tone bursts (duration, 1562 µs; envelope, Blackman; stimulation rate, 21.1/s) were delivered via an EC1 (electrostatic speaker – coupler model). The auditory evoked responses with 1024 sweeps were averaged. The auditory threshold was defined as the lowest sound intensity that evoked wave II [34].

The experimenters were blinded to the treatments of the rats. All cochleae were dissected immediately after ABR measurement on day 10. After dissection of the bony labyrinth, membranous labyrinth tissues were harvested from 18 rats (*n* = 6 per group). A total of 18 cochleae from 9 rats (*n* = 3 per group) were analyzed with quantitative reverse transcription-polymerase chain reaction (RT-PCR), and another 18 cochleae were analyzed by Western blotting. The remaining 12 cochleae from 6 rats were immersion-fixed in a 4% paraformaldehyde solution for histological examination.

### 4.2. Histological Examination

For hematoxylin and eosin (H&E) staining, paraffin blocks of decalcified cochleae were prepared as previously described [35]. Ten-micron-thick cochlear sections were sliced from the paraffin blocks using a rotary microtome. After mounting on glass slides, sections were deparaffinized, incubated in hematoxylin for 5 min, and stained with eosin for 45 s. Cochlear histological examinations were performed using an EVOS™ XL Core Imaging System (Invitrogen by Thermo Fisher Scientific, Waltham, MA, USA #AMEX1000). For cochlear whole mounts, cochlear outer hair cells (*n* = 2 per group for a total of 6 cochlea) were dissected after decalcification. The dissected cochlear outer hair cells were stained with 4′,6-diamidino-2-phenylindole (DAPI) as previously described and imaged using confocal microscopy [36].

### 4.3. Quantitative RT-PCR Analysis

Quantitative RT-PCR was conducted as previously described [37]. In brief, total RNA was extracted from cochleae using TRI Reagent^®^ (Sigma-Aldrich, St. Louis, MO, USA). TOPscript^TM^ RT DryMIX (dT18 plus; Enzynomics Co. Ltd., Daejeon, South Korea) was used for the reverse transcription reaction. Quantitative RT-PCR was performed with TOPreal™ qPCR 2 × PreMIX (SYBR Green with low ROX; Enzynomics, Daejeon, Korea) and oligonucleotide primers (Table 1) in a CFX96 Touch^TM^ Real-Time PCR Detection System (Bio-Rad, Hercules, CA, USA). mRNA expression levels were estimated based on the expression level of the reference gene *glyceraldehyde 3-phosphate dehydrogenase (GAPDH)*.

### 4.4. Western Blot Analysis

Western blot analysis was conducted as previously described [37]. In brief, radioimmunoprecipitation assay buffer (Cell Signaling Technology, Danvers, MA, USA) was used to lyse cochleae, and the protein concentration was measured using a Bio-Rad Protein Assay Kit (Bio-Rad, Hercules, CA, USA). Sodium dodecyl sulfate-polyacrylamide gel electrophoresis (8%) was performed to separate proteins, which were transferred to polyvinylidene difluoride membranes (Merck Millipore, Burlington, MA, USA). After soaking in blocking buffer (5% nonfat dry milk in Tris-buffered saline containing Tween-20 (TBS-T)) for 1 h, membranes were incubated with primary antibodies specific for AhR (mouse monoclonal, Santa Cruz Biotechnology, Santa Cruz, CA, USA, #SC-133088), HO1 (ADI-SPA-895, rabbit polyclonal, Enzo Stressgen, Farmingdale, NY, USA), SOD2 (ab13533, rabbit Immunoglobulin G (IgG), Abcam, Cambridge, United Kingdom), caspase 3 (rabbit polyclonal, Cell Signaling Technology, Danvers, MA, USA, #9662S), and β-actin (mouse monoclonal IgG_1_, Santa Cruz, Santa Cruz, CA, USA, #sc47778). Then, secondary antibodies (an anti-rabbit IgG horse radish peroxidase (HRP)-conjugated antibody (Cell Signaling Technology, #7074S) and a goat antimouse IgG heavy and light chain (H&L) HRP antibody (Abcam, #ab97023)) were applied. Immunoreactions were detected with an enhanced chemiluminescence kit (Bio-Rad, Hercules, CA, USA). Protein expression levels were estimated using ImageJ gel analysis software (National Institutes of Health, Bethesda, MD, USA) and compared with the expression level of beta-actin.

### 4.5. Statistical Analysis

ABRTs were compared among groups using repeated measures ANOVA with Tukey’s posthoc test for both pretreatment and post-treatment and for frequencies of 4, 8, 16, and 32 kHz. mRNA or protein expression levels were compared among groups using ANOVA with Tukey’s posthoc test. The values in the graphs are the means ± standard deviations (SDs). All analyses were conducted using SPSS software (ver. 21.0; IBM Corp., Armonk, NY, USA). Statistical significance was defined as a *p*-value of less than 0.05.

## 5. Conclusions

The present results establish the potential of ZO to attenuate CXP-induced auditory threshold shifts and inflammatory and apoptotic changes in cochleae. Because oxidative stress, inflammation, and apoptosis are shared mechanisms underlying ototoxicity caused by noise, aging, and aminoglycosides, ZO could be applied for various causes of ototoxic injuries. However, this study did not differentiate the mitochondrial expression levels of oxidative stress-related molecules from the whole-cell expression levels. In addition, clinical studies, including a toxicity analysis, should be performed before clinical use of ZO.

## Figures and Tables

**Table 1:** Oligonucleotide primer sequences used for quantitative reverse transcription-polymerase chain reaction.

Gene	Primer Sequence (Forward)	Primer Sequence (Reverse)	Annealing Temperature (°C)	Product Size (bp)	RefSeq Number
*CYP1A1*	5’- CATCCCCCACAGCACCATAA -3’	5’- TTCGCTTGCCCAAACCAAAG -3’	60	212	NM_012540.2
*CYP1B1*	5’- TGCTACTCGTTTCGGTCCTG -3’	5’- CAAGGCGAGCGAAGTACAAG -3’	60	162	NM_012940.2
*iNOS*	5’- AGGCCACCTCGGATATCTCT -3’	5’- TCTCTGGGTCCTCTGGTCAA -3’	60	85	NM_012611.3
*NFκB*	5’- TGTCTGCACCTGTTCCAAAGA-3’	5’- TGCCAGGTCTGTGAACACTC-3’	60	143	NM_199267.2
*IL6*	5’- AGAGACTTCCAGCCAGTTGC-3’	5’- TGAAGTCTCCTCTCCGGACT-3’	60	88	NM_012589.2
*TNFα*	5’- CGTCAGCCGATTTGCCATTT -3’	5’- TCCCTCAGGGGTGTCCTTAG -3’	60	88	NM_012675.3
